# Sensitivity analysis of Monte Carlo model of a gantry‐mounted passively scattered proton system

**DOI:** 10.1002/acm2.12803

**Published:** 2020-01-03

**Authors:** Milad Baradaran‐Ghahfarokhi, Francisco Reynoso, Michael T. Prusator, Baozhou Sun, Tianyu Zhao

**Affiliations:** ^1^ Department of Radiation Oncology Washington University St. Louis Missouri USA

**Keywords:** MCNP, Monte Carlo simulation, passively scattered proton, sensitivity analysis

## Abstract

**Purpose:**

This study aimed to present guidance on the correlation between treatment nozzle and proton source parameters, and dose distribution of a passive double scattering compact proton therapy unit, known as Mevion S250.

**Methods:**

All 24 beam options were modeled using the MCNPX MC code. The calculated physical dose for pristine peak, profiles, and spread out Bragg peak (SOBP) were benchmarked with the measured data. Track‐averaged LET (LET_t_) and dose‐averaged LET (LET_d_) distributions were also calculated. For the sensitivity investigations, proton beam line parameters including Average Energy (AE), Energy Spread (ES), Spot Size (SS), Beam Angle (BA), Beam Offset (OA), and Second scatter Offset (SO) from central Axis, and also First Scatter (FS) thickness were simulated in different stages to obtain the uncertainty of the derived results on the physical dose and LET distribution in a water phantom.

**Results:**

For the physical dose distribution, the MCNPX MC model matched measurements data for all the options to within 2 mm and 2% criterion. The Mevion S250 was found to have a LET_t_ between 0.46 and 8.76 keV.μm^–1^ and a corresponding LET_d_ between 0.84 and 15.91 keV.μm^–1^. For all the options, the AE and ES had the greatest effect on the resulting depth of pristine peak and peak‐to‐plateau ratio respectively. BA, OA, and SO significantly decreased the flatness and symmetry of the profiles. The LETs were found to be sensitive to the AE, ES, and SS, especially in the peak region.

**Conclusions:**

This study revealed the importance of considering detailed beam parameters, and identifying those that resulted in large effects on the physical dose distribution and LETs for a compact proton therapy machine.

## 1 INTRODUCTION

Proton therapy using compact passively scattered systems is undergoing a technological evolution as it eliminates the requirement for a complex beam transport system.^1^ However, additional absorbers are added to the gantry due to the lack of an energy selection system in a relatively short beamline. These absorbers significantly complicate the modeling of the system and justify careful characterization with precise and accurate methods to identify beam parameters, i.e. proton source that affect the uncertainty of calculated proton dose distributions.^2^

On the other hand, beside the proton physical dose distribution, its relative biological effectiveness (RBE) should also be taken into account.^3^, ^4^, ^5^ However, according to recent publications,^3^, ^6^ there is a significant variability in the RBE of protons as a function of depth or particle energy in the beam. In radiation dosimetry, linear energy transfer (LET) is one of the fundamental variables employed to derive the RBE.^7^ According to the recently published AAPM TG‐256, voxel‐by‐voxel dose‐averaged LET can be employed as a valuable tool for biologically optimized treatment planning even without knowing dose‐ and tissue endpoint‐specific RBE values accurately.^4^ Therefore, it is important to provide accurate proton LET distributions with rigorous sensitivity analysis, in addition to the physical dose, for clinical applications.

Monte Carlo (MC) simulation, as a gold standard tool in simulating complex radiation transport,^8^ plays an increasingly important role in proton therapy.^9^, ^10^, ^11^ Moreover, MC calculated LET values can be efficiently employed in the optimization of proton treatment planning systems (TPS) for clinical applications.^12^ Considering these advantages, MC simulation “can be an alternative or complimentary source of dosimetric data for developing, configuring, and validating analytical dose algorithms in clinical TPS”.^1^

In order to derive physical dose and LET distributions of a proton therapy unit by MC simulations, all major mechanical components of the treatment nozzle should be modeled in detail.^9^, ^13^, ^14^ However, even detailed simulation of all machine components cannot account for deviations, especially for the radiation source, from factory specifications.^11^, ^15^, ^16^ Furthermore, the source information provided by the manufacturer is often limited to spot size and nominal energy, and customization of the MC model is required to match its results with the measured data.

The large number of adjustable parameters in a clinical proton therapy system (e.g., average energy, energy spread, spot size, beam offset from central axis, etc.) demands a thorough sensitivity analysis that provides important characteristics that are difficult or impossible to measure. In addition, the routine quality assurance processes can be significantly facilitated by correlating the adjustable simulation parameters with measured dose distributions.

In this study, physical dose and LET distributions of a passive double scattering compact proton therapy unit, known as Mevion S250 (Mevion Medical Systems, Littleton, MA, USA), were calculated using the Monte Carlo N‐Particle eXtended (MCNPX) MC code. The physical dose results were benchmarked with measured commissioning data. Additionally, a sensitivity analysis of the Mevion S250 unit was done by adding small variations of treatment nozzle and proton source parameters and characterizing their impact on the depth of the pristine peak, shape, and symmetry of the resulting dose profiles, and LET distribution in a water phantom.

The aim of this work is to present guidance on the correlation between treatment nozzle and proton source parameters, and physical dose distribution to the following: for researchers modeling clinical proton beam systems, and clinical medical physicists tasked with physically tuning their passive double scattering compact proton therapy unit to bring beam parameters to within clinically acceptable levels. Moreover, this work proposes to create a reference library to troubleshoot of the machine installed and commissioned in the S. Lee Kling Proton Therapy Center at Barnes‐Jewish Hospital in St. Louis, MO, USA.

To the best of our knowledge, this is the first reported simulation and sensitivity analysis of the Mevion S250 using MCNPX. The Mevion S250 machine is particularly noteworthy because of its unique beam characterization, which is due to the lack of energy selection and beam transportation systems, and mounting interest in single‐room proton unit.^17^

## 2 MATERIALS AND METHODS

### 2.1 MCNPX MC simulation of Mevion S250

#### 2.1.1 Physical dose

**Figure 1:**
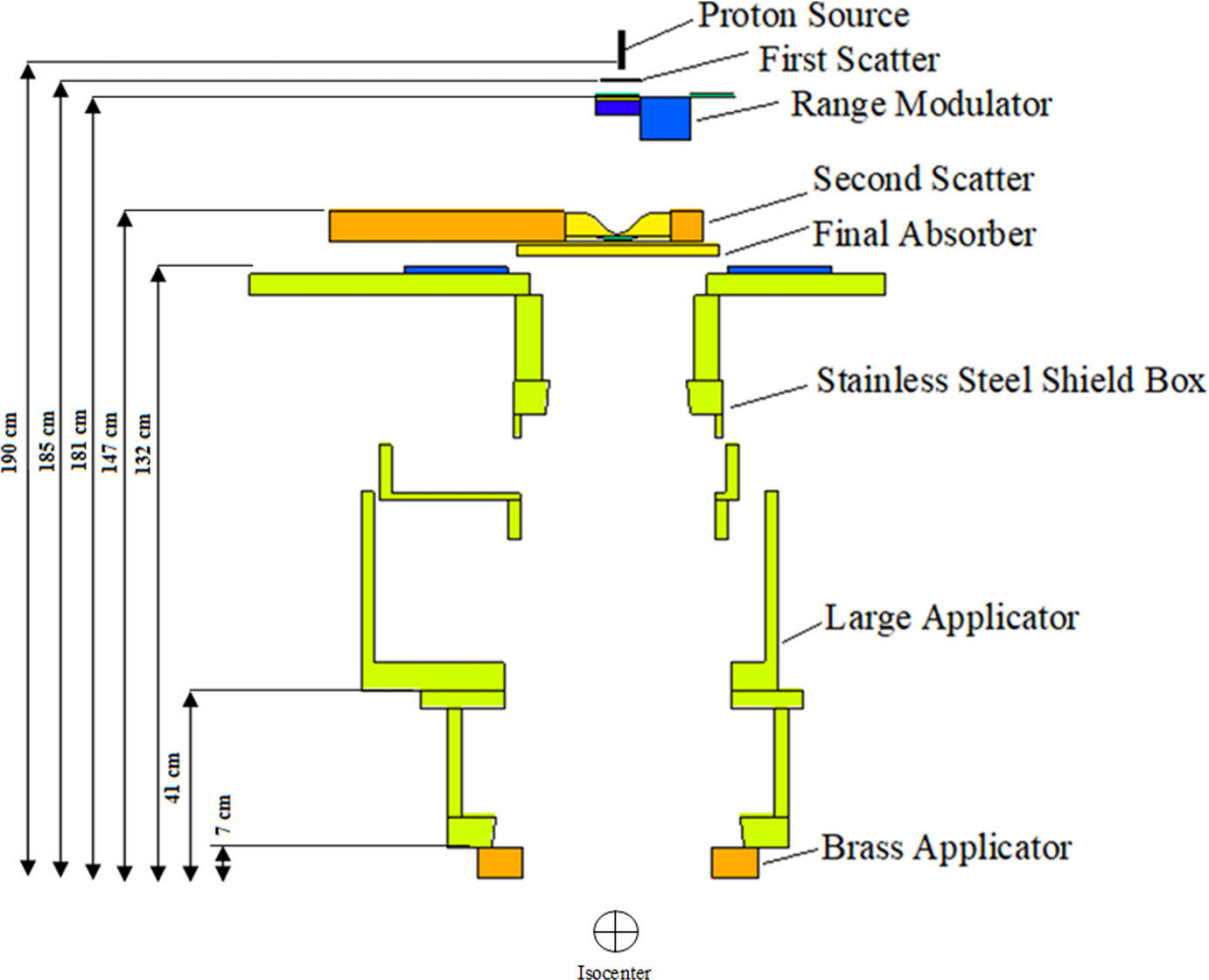
A cross‐section view of the simulated Mevion S250 treatment nuzzle for the deep group (distance from the proton source to the isocenter was 205 cm).

The Mevion S250 has 24 different beam options divided into large, deep, and small groups.^2^ Each beam configuration is generated using a unique arrangement and combination of different beam line components (Fig. 1). In order to acquire physical dose distribution data for each configuration, three sets of measurements are taken: (a) pristine peak, (b) lateral profiles in air, and (c) spread out Bragg peak (SOBP).

To obtain pristine peaks and SOBPs, a parallel‐plate chamber (PPC05, IBA Dosimetry) was used to measure percent depth‐dose curves in a 3D scanning tank (Blue phantom, IBA Dosimetry America, Bartlett, TN, USA) at nominal source‐to‐surface distance (SSD) of 200 cm with radiation isocenter placed on the water surface. Lateral profiles in air were measured using a diode Edge detector (Sun Nuclear Corporation, Melbourne, FL, USA). The general guideline for acquiring beam data for photon machines has been described in the report of AAPM Task Group 106.^18^

For the MC simulations, MCNPX was used in this work.^19^ Computations were performed using the facilities at the Washington University, Center for High Performance Computing. The simulation was started by using the manufacturer’s specifications for all dimensions and materials of each beam component. Then, average energy, energy spread (FWHM), spot size, and First Scatter (FS) thickness were tuned to match the measured data (experimental results).

For SOBP simulations, a user defined beam current modulation (BCM) sequence was defined considering the rotation angle of the range modulator wheel.^10^, ^20^ According to the method described by Polf et al. individual pristine Bragg peaks were created and weighted to form a uniform and flat SOBP with the desired modulation.^10^

For the pristine peak and lateral profiles, for each dose point, MCNPX derived data were compared with the measurements by calculating the local difference. In addition, for the penumbra region of the profiles, distance between the 80% and 20% dose levels was compared. SOBP was evaluated by comparing the simulated results and measured data, on the SOBP width as defined by the proximal 95% to the distal 90% dose, beam range, and the depth of distal 20% dose. The beam range was defined as the depth of 90% dose (D90%) on distal fall‐off.

The simulations in water and air were performed with 2.0 × 10^9^ and 7.5 × 10^8^ histories respectively. Generally, for the so‐called “good practice” in MC simulations, enough history should be calculated to ensure that MC results have at least 1σ (k = 1, 67% confidence index) <1% statistical uncertainty at depths of interest in the water and air.^19^

The mesh tally detector was used due to its functionality for proton dose calculations.^19^ The photons, electrons, protons, and positrons were suppressed for simulations with a cutoff energy of 990 eV, 57.3 keV, 5 keV, and 56.6 keV respectively.

For comparison purposes, the results were then compared with the derived values from TOPAS (version 2.0) simulations.^20^

#### 2.1.2 LET

After tuning the treatment nozzle for all the 24 options, both track‐averaged LET_t_ and dose‐averaged LET_d_, were calculated according to the method by Guan et al.^7^ It should be noted that, LET_t_ calculated as the arithmetic mean value of the fluence spectrum, matches the definition by the ICRU, and LET_d_ is a quantity that accounts for both physical dose and LET, to predict biological effects.^7^, ^19^

To calculate both LET_t_ and LET_d_, the detector cells were modeled as spheres on the central axis of the beam in water phantom. The MCNPX LET special tally was employed to record flux over the cells as a function of stopping power instead of energy.^19^ Using this tally, the recorded values in the energy bins are interpreted as stopping power values (units of MeV/cm).

## 3 SENSITIVITY ANALYSIS

The sensitivity of the model to changes in machine proton source parameters was analyzed by varying the Average Energy (AE), Energy Spread (ES), Spot Size (SS), Beam Angle (BA), and Offset from central Axis (OA). FS thickness was the only treatment nozzle parameter used for the sensitivity investigations. In this step, all 24 options of Mevion S250 were simulated in different stages to obtain the uncertainty of the derived results on the depth of the pristine peak, shape, and symmetry of the resulting dose profiles, as well as LET_t_ and LET_d_ distributions. For the depth of pristine peak, the dose distribution was evaluated using the distal 90% (D90%). Because, each SOBP was created by superimposing single Bragg peaks, the sensitivity study of the pristine peaks also reflects uncertainties associated with SOBPs.^10^, ^13^, ^15^ The flatness and symmetry of the profiles were analyzed using the method proposed by Prusator et al.^20^ as follow:

(1) $$Flatness=\left(D_{\min}-\;D_{\max}\right)/\left(D_{\min}+\;D_{\max}\right)*100$$

where, *D*_min_ and *D*_max_ are, respectively, the minimum and maximum doses within the central axis of the beam to the 80% dose levels.

(2) $$\begin{array}{c} Symmetry\;=\;\left(LD_{\text{int}egral}-\;RD_{\text{int}egral}\right)/\left(LD_{\text{int}egral}+\;RD_{\text{int}egral}\right)*100 \end{array}$$

where, *LD_integral_* and *RD_integral_* are the integral doses of the left and right side of the radiation field respectively.

The stages included in the sensitivity study were the AE (±9%), the ES (FWHM) [up to +20%], SS (up to +7 mm), BA (0°–2°), OA, and SO (up to +15 mm), and increasing FS thickness (10%). The variations in each stage revealed the potential uncertainties in these parameters. These uncertainty values were chosen based on clinical operation, representing the likely extent of adjustments required to bring a system to within acceptable tolerances.

Since for each group (large, deep, and small), treatment nozzle configurations are very similar in design, in this study we will only refer to the group, instead of the option, for the sensitivity analysis.

## 4 RESULTS

With the number of simulated histories used, the uncertainty associated with statistical (quantum) uncertainty in the MC‐calculated results in air was less than 0.50% at all distances in the transverse plane. For the large group, the statistical uncertainty for calculations in water was less than 0.61% for the depths of 25.0 cm (D90% of the deepest Bragg Peak). Whereas, for the deep and small groups, it was 0.94% and 0.69% for D90% of 31.9 and 20.0 cm, respectively.

This statistical uncertainty made it feasible to investigate noticeable effects on the physical dose distribution due to slight changes of the sensitivity study parameters. In other words, high precision was obtained in the results of the simulations, which was due to simulating large numbers of histories.

### 4.1 Physical dose and LET

For the pristine peak, the MCNPX MC model of the Mevion S250 matched measurements data for all the 24 options to within 2% of the dose points compared and 1 mm for the distances to agreement (within 2% or 1 mm criterion). Table 1, shows comparison of derived percent depth dose data with MCNPX, for the large, deep, and small options, with measurements and TOPAS simulations for this system. Comparing the MCNPX and TOPAS results, there were differences of up to 1.4% in the derived DD_0.5 cm_, the largest discrepancies occurring in the deep options.

**Table 1:** Comparison of derived percent depth dose data with MCNPX, for the large, deep, and small options, with measurements and TOPAS simulations.^20^ Data are presented for dose at depth of 0.5 cm (DD_0.5 cm_) and 3.0 cm (DD_3.0 cm_) and the beam range, defined as the depth of 90% dose (D90%) on distal fall‐off.

Option		∆DD_0.5 cm_ (%)	∆DD_0.5 cm_ (%) [TOPAS]	∆DD_3.0 cm_ (%)	Measured D90% (cm)	∆^a^ in D90% (cm)	∆ in D90% (cm) [TOPAS]
Large group	1	1.4	0.8	1.0	24.95	0.02	0.04
	2	0.7	0.8	0.6	22.63	0.03	0.05
	3	1.1	0.4	1.1	20.96	–0.06	‐0.05
	4	1.5	0.5	1.6	18.82	0.04	0.07
	5	0.7	1.1	0.5	16.84	0.05	0.06
	6	1.7	1.5	1.4	14.87	–0.05	0.04
	7	0.8	1.0	1.7	13.16	0.04	0.05
	8	0.6	0.6	0.6	11.45	0.04	‐0.09
	9	1.6	1.2	1.4	10.07	0.04	0.05
	10	1.2	0.5	1.2	8.66	0.03	0.09
	11	0.2	1.5	0.4	7.35	0.04	0.08
	12	1.7	1.3	1.6	6.13	0.05	0.09
Deep group	13	1.9	1.2	1.5	31.95	0.03	0.04
	14	0.4	0.6	0.5	29.48	0.04	0.00
	15	1.1	1.6	0.8	26.94	0.03	0.02
	16	0.4	1.8	0.4	24.49	0.04	0.03
	17	0.9	1.1	1.2	22.12	0.04	0.04
Small group	18	1.4	1.5	1.3	20.06	0.03	0.05
	19	0.4	1.9	0.6	17.81	–0.06	0.07
	20	1.3	0.3	1.3	15.43	0.03	0.07
	21	1.6	1.5	0.8	13.36	0.02	0.02
	22	0.8	1.7	1.3	11.18	–0.03	0.04
	23	1.1	0.6	1.0	9.10	–0.05	0.00
	24	0.3	1.2	0.6	7.01	–0.05	0.00
Avg. (Max)		1.03 (1.9)	1.09 (1.9)	1.01 (1.7)		0.039(0.06)	0.048 (0.09)

a Discrepancy (D90% _Measurement_ – D90% _MCNPX Simulation_).

**Figure 2:**
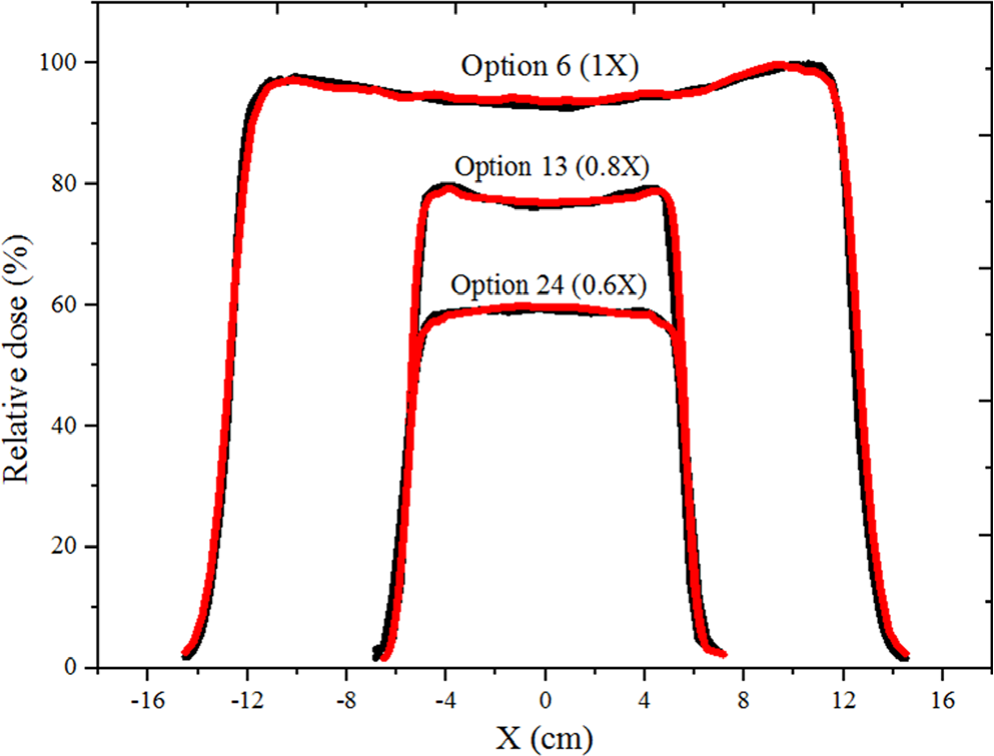
Comparison between the MCNPX derived (red) and measured (black) lateral beam profiles for options 6 (large group), 13 (deep group), and 24 (small group). The lateral beam dose profiles for options 13 and 24 are multiplied by 0.8 and 0.6, respectively, to show all three comparisons in one graph.

Fig. 2 illustrates a comparison between the derived lateral beam profiles in this study with the measured values, where only the data for options 6, 13, and 24 are presented for the sake of brevity. The largest dose discrepancy between MC calculations and measurements was 2% and in the penumbra region, whereas, between the 80% and 20% dose levels the distance‐to‐agreement was less than 1.0 mm. For the TOPAS simulations, the absolute differences in the penumbras between simulated and measured profiles at each depth for each configuration all agreed to well within 0.6 mm.

Flatness and symmetry of the profiles for the large group were less than 1.44% and 0.40%, respectively, whereas, for the deep group they were 1.18% and 0.30%, respectively. Small group showed the lowest flatness and symmetry (2.18% and 0.60%, respectively).

For all the SOBPs, the distal 90% and 20% depths were matched with measurements within 1.0 and 1.7 mm differences, respectively. For width of the created SOBPs, the largest discrepancy was less than 2.0 mm compared to the experimental measurements.

Based on the treatment nozzle benchmarking results, for all the 24 options, the primary proton source energy was finally set to be 252 MeV with an initial Gaussian distribution profile in energy (σ_E_ = 0.40 MeV) and in space (σ_x_ = σ_y_ = 2.7 mm). The nominal energy provided by the vendor was 250 MeV and the energy spread was 0.4% (RMS).

**Figure 3:**
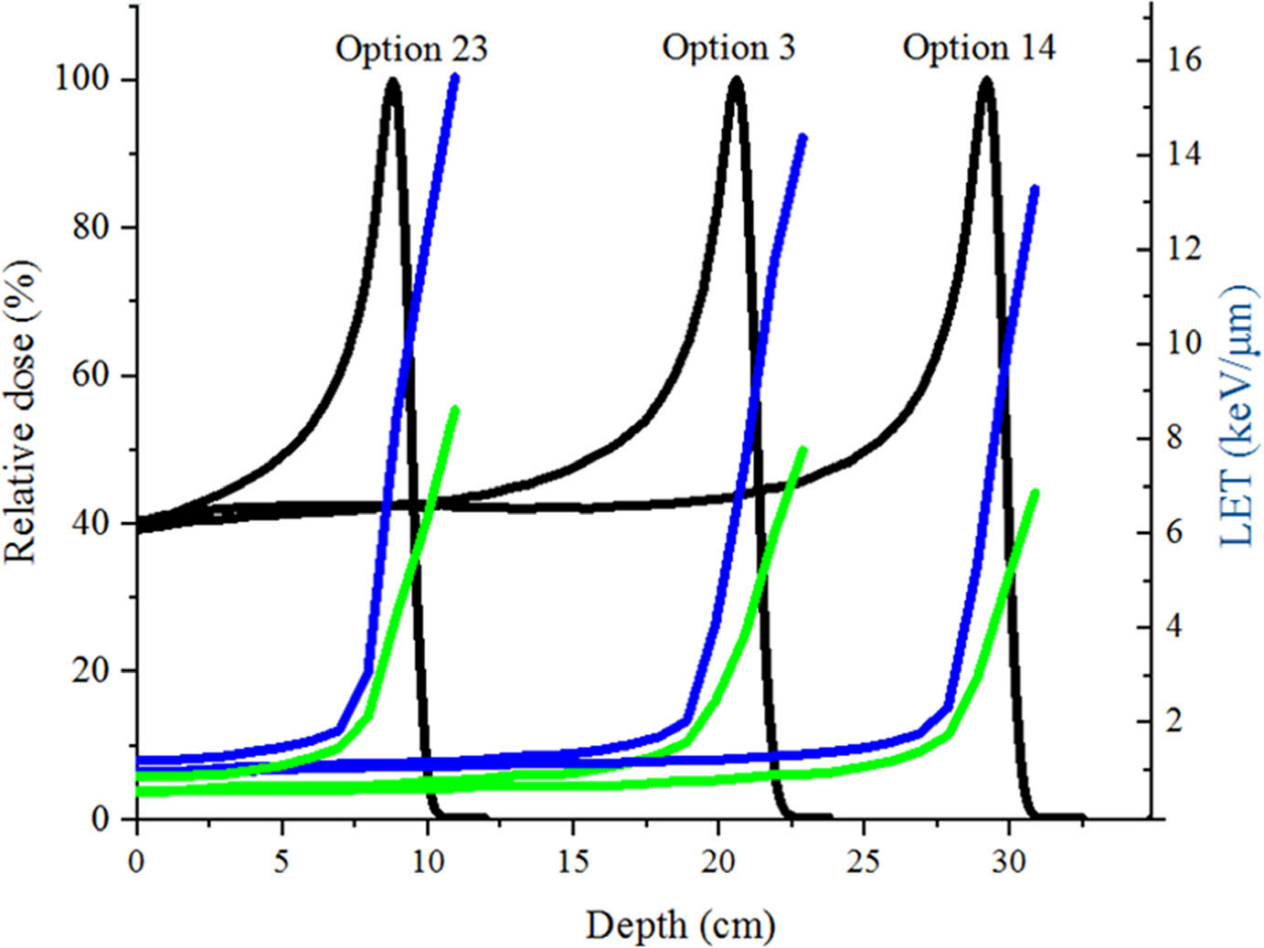
Distributions of LET_t_ (green) and LET_d_ (blue) as a function of depth for each main Bragg peak of the options 3 (large group), 14 (deep group), and 23 (small group).

The Mevion S250 was found to have a LET_t_ between 0.46 and 8.76 keV.μm^–1^ and a corresponding LET_d_ between 0.84 and 15.91 keV.μm^–1^. The distributions of LET_t_ and LET_d_ as a function of depth for options 3, 14, and 23 are shown in Fig. 3. After the distributions of LET_t_ and LET_d_ for each option were established for a single Bragg peak, it was easy to superimpose several LET_t_ and LET_d_ for more complex situations, according to the defined BCM for each Bragg peak.^21^

### 4.2 Sensitivity analysis

#### 4.2.1 Average energy

In this study, using the 250 MeV nominal beam energy of the system, the predicted D90% was within 3 mm for the small group, compared to the experimental measurements. For the large and deep groups, even less discrepancy (<2 mm) was seen.

**Figure 4:**
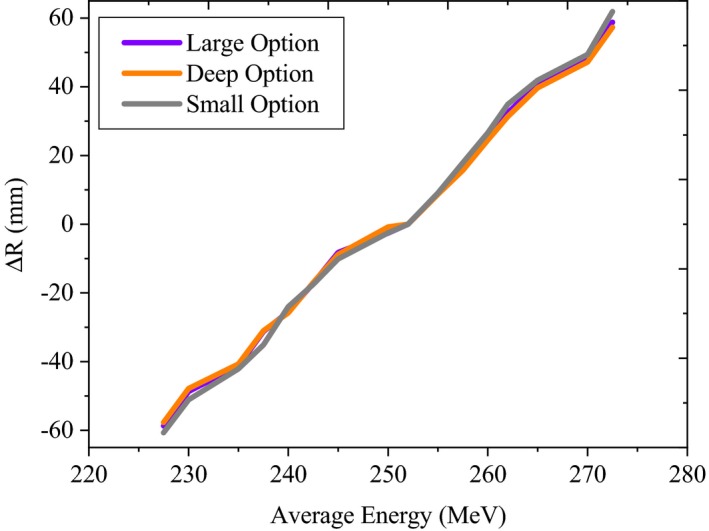
The absolute difference (ΔR) between the baseline and MC derived depth of D90% as a function of changes in the AE for large, deep, and small groups.

Fig. 4, illustrates the absolute difference between the measured and MC derived depth of D90% as a function of changes in the AE for each group (large, deep, and small). As expected, there were differences among these groups since each group uses a unique treatment nozzle configuration. Slight changes in the AE (3%) significantly influenced the D90% (up to 26.6 mm) for small group. Whereas, for the large and deep groups, up to 23.4 and 22.1 mm, respectively, changes of the D90% was observed. Moreover, the deep group was less sensitive to 9% AE uncertainty (up to 57.3 mm), compared to the large (58.8 mm) and small groups (61.1 mm).

**Figure 5:**
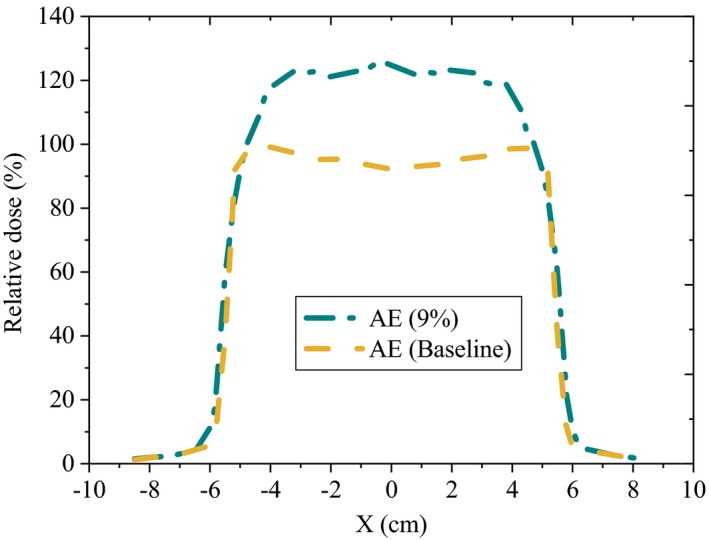
Variations of the flatness of lateral profile for small groups due to 9% increase in the AE.

Altering the proton beam energy to 109% showed a strong effect on the small group profile flatness (up to 8.6%), whereas results on the symmetry of the profiles were not significant (less than 2.2%) and remained within the statistical uncertainty of the MC calculations. Figure 5, demonstrates the variations of the flatness and symmetry of lateral profiles for the small group due to 9% increase in the AE.

#### 4.2.2 Energy spread (FWHM)

**Figure 6:**
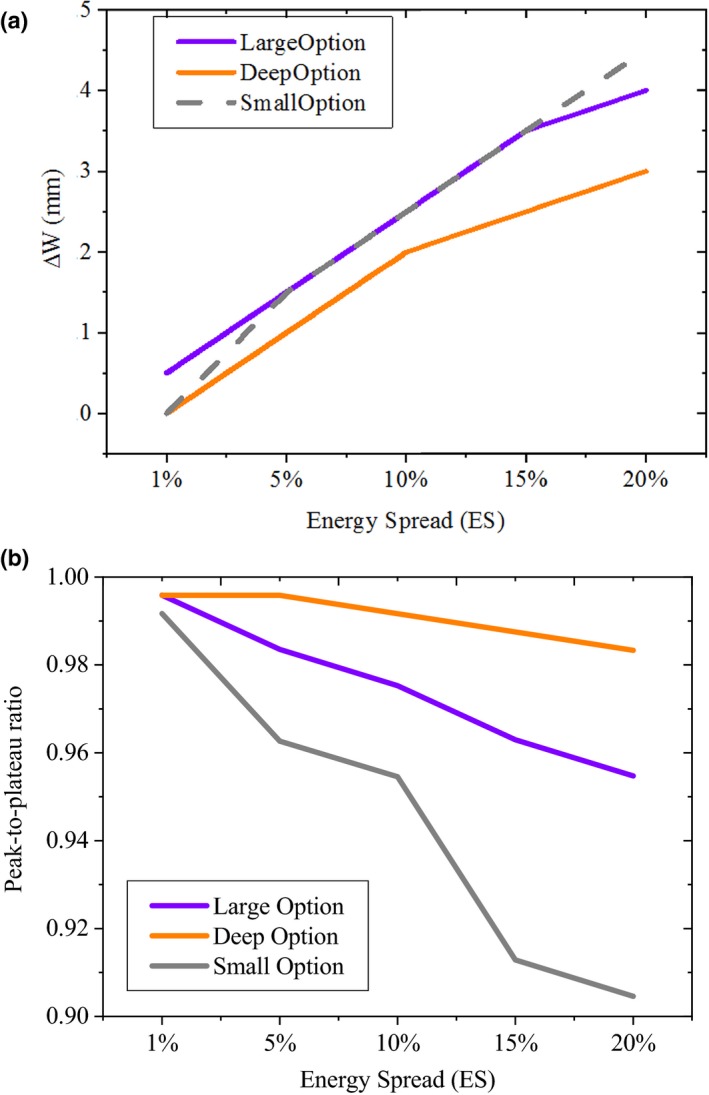
Absolute difference between the baseline and sensitivity derived width of the pristine peaks (a) and peak‐to‐plateau ratios (b) due to change in the ES for the studied groups (large, deep, and small). Peak‐to‐plateau ratio was derived based on the ratio of the peak‐to‐plateaus of sensitivity results to the baseline values (peak‐to‐plateau_Sensitivity_/peak‐to‐plateau_Baseline_).

Figure 6, shows the absolute difference between the baseline and sensitivity derived width of the pristine peaks and peak‐to‐plateau ratios (peak‐to‐plateau_Sensitivity_/peak‐to‐plateau_Baseline_) of the studied groups (large, deep, and small). Due to 20% increase in the ES, the maximum increase in the width of the pristine peaks were 4.1 and 2.9 mm for the large and deep groups respectively, whereas, the maximum decrease in the peak‐to‐plateau ratio were 4.5% and 1.6%, respectively. For the small group, the maximum differences for the width of the pristine peaks and peak‐to‐plateau ratios were 4.4 mm and 9.5%, respectively.

#### 4.2.3 Spot size

**Figure 7:**
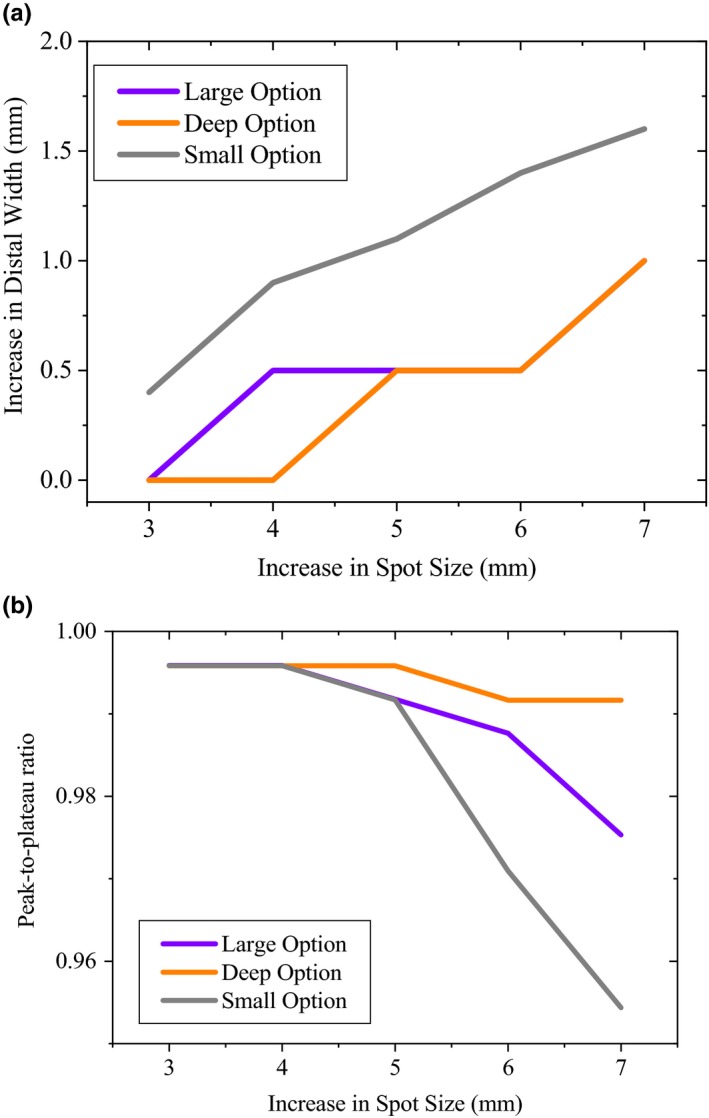
The increase in distal width and decrease in peak‐to‐plateau ratio of the pristine peaks due to uncertainty associated with SS in large, deep, and small groups. Increase in distal width was calculated based on the absolute difference of the baseline and sensitivity derived results. Peak‐to‐plateau ratio was derived based on the ratio of the peak‐to‐plateaus of sensitivity results to the baseline values (peak‐to‐plateau_Sensitivity_/peak‐to‐plateau_Baseline_).

It was revealed that changes of the SS resulted in no significant effect on the pristine peaks D90% (<1 mm). Figure 7 gives the changes of distal width and peak‐to‐plateau ratio of the pristine peaks due to uncertainty associated with SS in large, deep, and small groups. It was observed that, considering up to 3 mm uncertainty of the SS, there was no significant effect on the distal width (<1 mm) and peak‐to‐plateau ratio (<1%) owing to the beam spot size change. In our study, the distal width of a Brag peak was defined as the difference between the 10% and 90% dose on the distal fall‐off. Noticeable effect on the distal width (about 2 mm) was seen for SS of 10 mm, especially for the small group (Fig. 7).

Therefore, the beam SS have little influence on pristine peaks and the effects might be too subtle to produce significant changes to the distal width of the dose distribution.

Varying the proton beam SS resulted in negligible effect (<1%) on the flatness and symmetry of the small group. The changes for the large and deep groups were also remained approximately within the statistical uncertainty of the calculations.

#### 4.2.4 Beam angle

**Figure 8:**
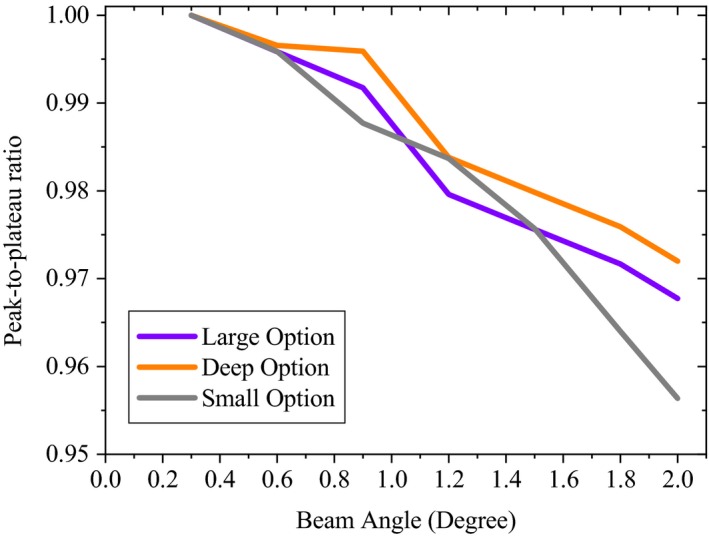
Variations of the peak‐to‐plateau ratio due to changes of the incident angle of the proton beam. Peak‐to‐plateau ratio was derived based on the ratio of the peak‐to‐plateaus of sensitivity results to the baseline values (peak‐to‐plateau_Sensitivity_/peak‐to‐plateau_Baseline_).

Figure 8 shows that the 2° incident angle of the proton beam had a significant effect on the peak‐to‐plateau ratio (up to 4.5% decrease) of the Bragg peaks, whereas, the effects on the depth of D90% were small (<1 mm).

On the other hand, a 0.6° deviation from normal was found to change the flatness by 2.7% for small group and 1.8% for deep group (Table 2). The maximum effect was seen for the small group (up to 7.6%) for a 2.0° proton beam deflection. The symmetry was less affected by the BA, as to see a 2.0% decrease in the flatness of the profiles; the proton beam should be deflected by a minimum of 0.6° for the small option. For the large and deep options, more beam deflection was needed (>0.9°) to reproduce a same effect. The 2% criterion for the flatness and symmetry was selected to present the likely level of adjustments needed to bring a proton system to within acceptable clinical specifications. Moreover, it may be of an interest for tuning a MC simulation of clinical proton systems.

**Table 2:** Variations of the flatness and symmetry of the profiles due to changes of the incident angle of the proton beam.

BA (°)	Flatness (%)			Symmetry (%)		
	Large opt.	Deep opt.	Small opt.	Large opt.	Deep opt.	Small opt.
0.3	1.88	2.05	3.44	1.0	1.10	1.54
0.6	3.1	2.38	4.17	1.38	1.41	1.82
0.9	4.32	2.66	4.65	1.74	1.54	2.14
1.2	4.78	3.2	5.13	2.27	2.05	2.45
1.5	5.65	4.34	6.43	2.48	2.36	2.77
1.8	6.89	5.76	7.15	2.59	2.47	3.01
2.0	8.5	6.78	8.78	2.85	2.58	3.52

#### 4.2.5 Offset from central axis

**Figure 9:**
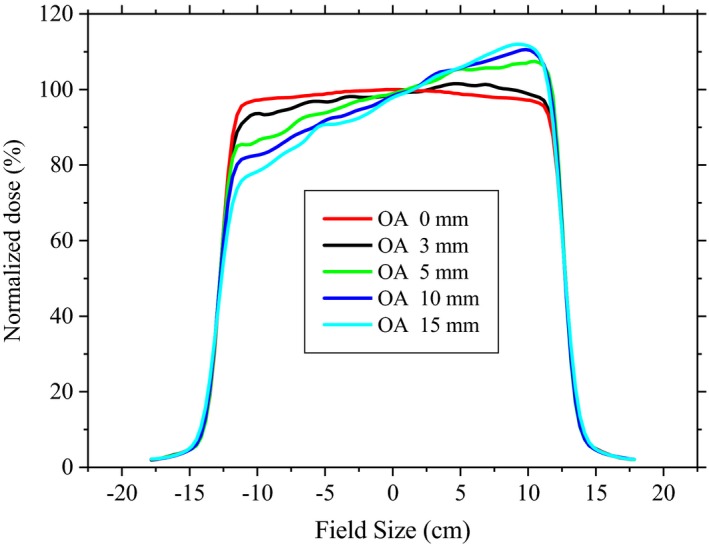
Variations of the flatness and symmetry of the large group profiles due to uncertainty in the OA.

**Figure 10:**
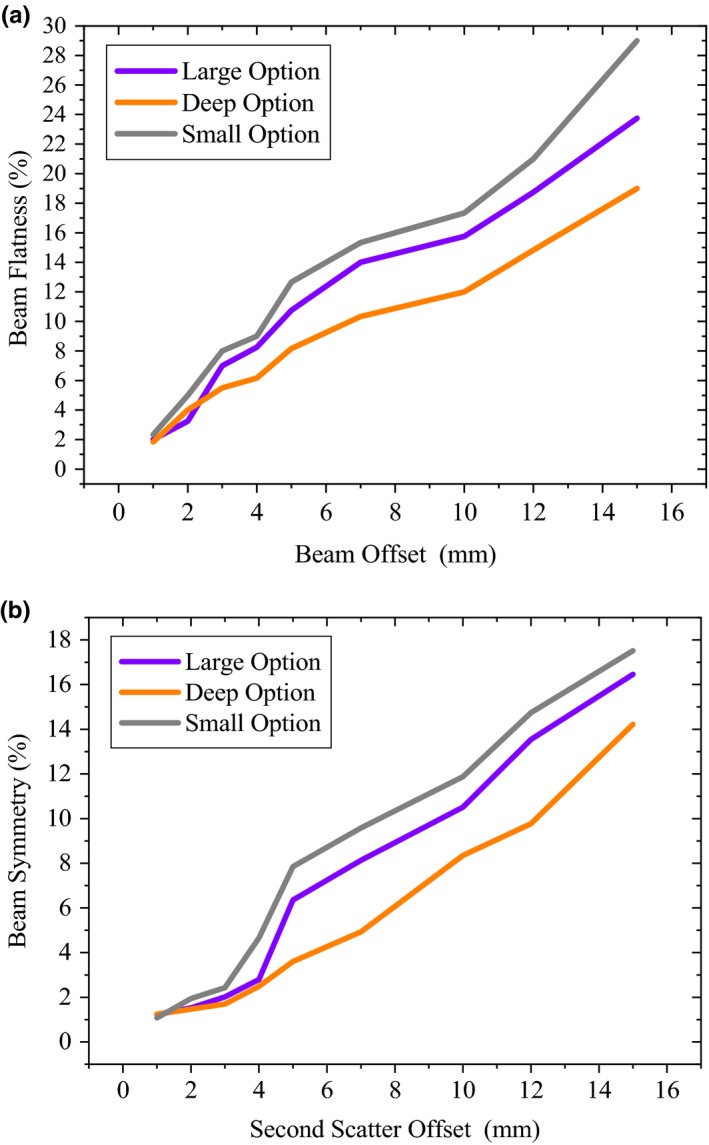
Changes in the flatness (a) and symmetry (b) of the profiles due to uncertainty associated with second scatter offset from the central axis.

For a given lateral offset of 3 mm, the beam flatness and symmetry were significantly affected, more strongly, for small (3.7% and 3.3%) and large (3.5% and 2.8%) groups, compared to the deep group (2.4% and 2.4%). As expected, increasing the OA for up to 15 mm significantly decreased flatness and symmetry of the profiles, especially for small option (31.0% and 11.4%). Figure 9 gives variations of the flatness and symmetry of the large group profiles due to uncertainty in the OA. Similar results were observed for the SO. In other words, considering the statistical uncertainty of the MC results, OA was indistinguishable from SO. Figure 10 shows changes in the flatness and symmetry of the profiles due to uncertainty associated with SO from the central axis.

Moreover, 3–15 mm OA resulted in change of D90% (up to 3 mm). According to our results, small groups was found to be more sensitive on the OA and SO small changes compared to the other treatment nozzle configurations.

#### 4.2.6 First Scatter thickness

In this work, it was hypnotized that changes in the thickness of the lead FS have a much more prominent effect on the depth of D90%. The maximum thickness of the FS is 8.370 mm (large group‐ option 12) to 1.322 mm (deep group‐ option 17) depending on the treatment nozzle configuration. Therefore, options 1 and 13 with corresponding FS thickness of 6.167 mm and 1.322 mm, respectively, were selected for the sensitivity analysis.

**Figure 11:**
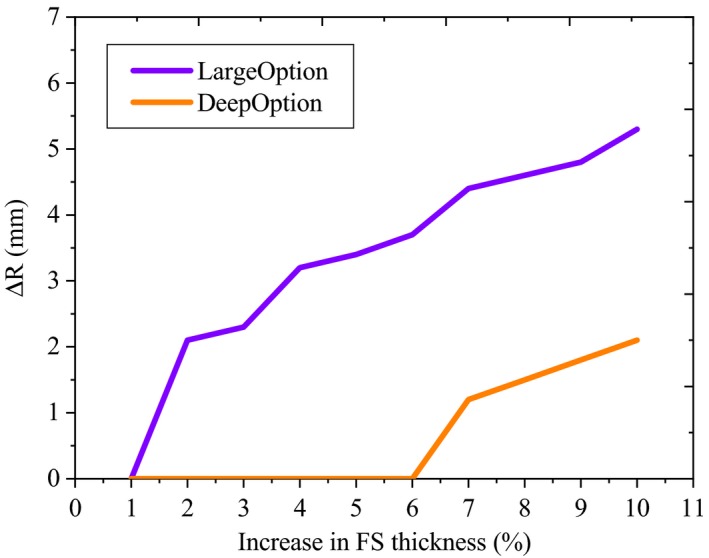
Changes of the depth of D90% as a function of FS thickness for large and deep options.

For the small group, there is no FS in the treatment nozzle configuration; therefore, we were unable to investigate the sensitivity of the derived dose distribution due to uncertainty in FS thickness. Figure 11 gives the changes of the depth of D90% as a function of FS thickness for large and deep options. Changes in FS thickness affect the range, especially for the large group (up to 5 mm).

Increasing the FS thickness within the tolerances in this study (up to 10%), had a comparatively minor effect on the profile flatness and symmetry (less than 2%).

#### 4.2.7 LET

**Figure 12:**
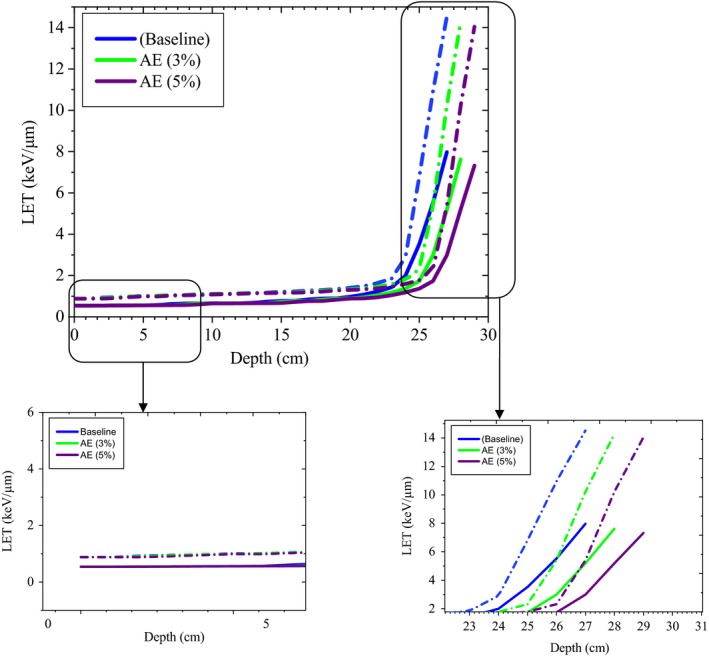
Changes of the LET_d_ (dash line) and LET_t_ (solid line) distributions due to increase in the AE for the large group.

For the small group due to 9% increase in the AE, in the peak region the LET_d_ decreased by up to 2.03 keV/μm^−1^ representing 12.8% of its maximum value, whereas LET_t_ was decreased by up to 1.59 keV/μm^−1^ (14.6% of its maximum value). In the plateau region, LET_d_ and LET_t_ were less affected (up to 4.9% and 2.7%, respectively). In other words, LET_t_ was less sensitive than the LET_d_ by up to 2.2%. For this group, the plateau maximum values of the LET_d_ and LET_t_ were 0.89 keV/μm^−1^ and 0.54 keV/μm^−1^ respectively. For the small group, slight changes in the AE (<3%) did not significantly influence the LETs (less than 2%). Our results showed that, deep group was less sensitive in the both peak (<4.7%) and plateau (<2%) regions compared to the small group. For deep group, the maximum values of the LET_t_ and LET_d_ were 6.97 keV/μm^−1^ and 13.42 keV/μm^−1^ respectively. The maximum values of the LET_d_ and LET_t_ in the plateau were 0.84 keV/μm^−1^ and 0.46 keV/μm^−1^ respectively. Figure 12, illustrates changes of the LET_t_ and LET_d_ distributions due to 3% and 5% increase in the AE for the large group.

**Figure 13:**
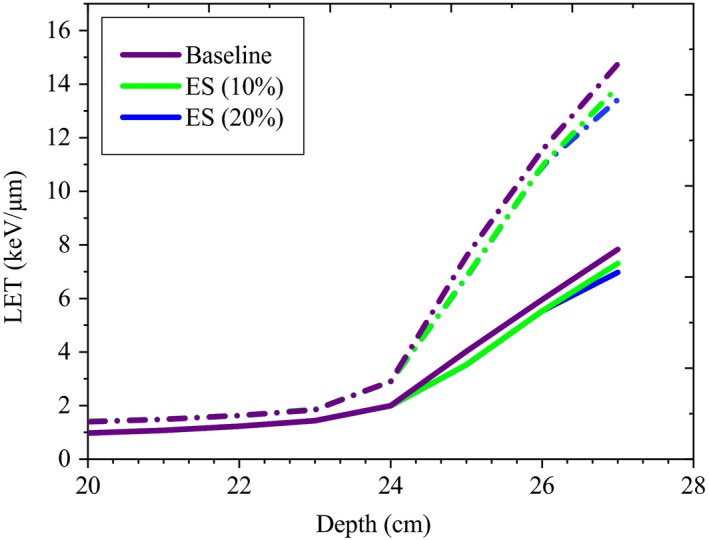
Changes of the LET_d_ (dash line) and LET_t_ (solid line) distributions due to increase in the ES for the large group.

As ES becomes larger (up to 20%), for all the groups, the absolute maximum of LET_d_ and LET_t_ become lower (up to 1.67 keV/μm^−1^ and 1.23 keV/μm^−1^, respectively), and less steep at the end of the range (Fig. 13). Increasing the ES resulted in decrease in LET_t_ and LET_d_ for the peak regions, especially for the small group (up to 9.2% and 9. 6% respectively). For the deep group, decrease in the LET_t_ and LET_d_ was remained within less than 1% of their maximum values. Both LET_t_ and LET_d_ in the plateau region were even less sensitive (<1%) to changes in the ES within the uncertainties studied in this work. Figure 13, shows the variations in the LET_d_ (dash line) and LET_t_ (solid line) distributions due to increase in the ES for the large option.

**Figure 14:**
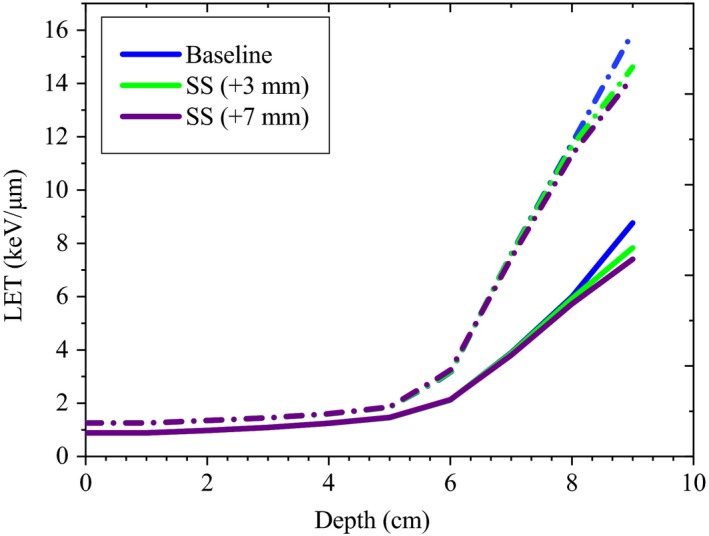
Variations of the LET_d_ (dash line) and LET_t_ (solid line) distributions due to increase in the SS for the small group.

Due to 7 mm increase in the SS, the maximum decrease in the LET_d_ and LET_t_ for the large group was up to 0.56 keV/μm^−1^and 0.34 keV/μm^−1^, respectively, representing 4.1% and 4.8% of their maximum value. For this group, up to 5 mm increase in the SS, resulted in slight changes of the LETs (<2%). For the deep group, changes were within 1% of their maximum values. For small and large groups, both LET_d_ and LET_t_ were less steep at the end of the range (Fig. 14). Figure 14, shows variations of the LET_t_ and LET_d_ distributions due to increase in the SS for the small group. For all the groups, similar to ES, changes in the plateau region were not significant (<1%).

## 5 DISCUSSION

There is no literature available on the sensitivity analysis of physical dose and LET distributions of Mevion S250 as a passive double scattering compact proton therapy unit.

The MCNPX benchmarked physical dose with the measured data showed up to 2% or 1 mm discrepancy (Table 1; Fig. 2), mainly due to the physical properties of various materials^15^ in the treatment nozzle as well as uncertainty associated with the cross section library,^22^, ^23^ which were not evaluated in this work. However, it has been stated that the default configurations, i.e. cross section library, of the MCNPX MC code lead to results that are within to the clinically used quality assurance criteria.^24^

Results of the MCNPX simulation were in reasonably good agreement with the TOPAS simulations (<2% of the dose points compared and <1 mm for the distances) (Table 1). Small differences between the MCNPX and TOPAS, especially for the lateral beam profiles, may be due to the shape of the aperture cut‐out, which was relevant for penumbra region and was more difficult to model accurately in MCNPX. Zhao et al. observed noticeable differences along the field edges, both inside and outside of the fields, at shallow depths. They stated that, the differences were significantly decreased with increasing depth in water.^2^

MCNPX model sensitivity analysis for all the options revealed that, the AE and ES had the greatest effect on the D90% and peak‐to‐plateau ratio (Figs. 4, 5, 6) respectively. Although, the AE of a proton beam is not as of clinical interest as the range, since the range can be measured very accurately,^15^ it should be known as starting points for the MC simulations. The ES can provide one of the largest sources of uncertainty in simulation due to the difficulty in measuring it. Similar to our findings, it has been reported that, the finite width and peak‐to‐plateau ratio of the Bragg peak is very sensitive to the ES of the proton beam.^13^, ^15^ It should be noted that, for a proton system, the relationship between AE and ES is not known accurately. Our results were in a good agreement with Paganetti et al.^13^ who simulated the Francis H Burr proton beam treatment nozzle, at the Northeast Proton Therapy Center at Massachusetts General Hospital, to aid in the commissioning process and support clinical operation. They found that, increasing the energy spread results in the broadening of the Bragg peak and consequently decrease in the peak‐to‐plateau ratio. Bednarz et al. stated that change in beam energy spread does not have a noticeable effect on the uniformity of the SOBP.^15^ We found that, for the Mevion S250, variations of the proton ES had no significant effect on the flatness and symmetry of the beam profile for the large and deep groups. For the small group, up to 2.6% decrease in the beam flatness was observed due to 20% change in the ES.

On the other hand, it has been reported that, the beam SS may influence the peak‐to‐plateau ratio of pristine Bragg peaks, and accordingly the SOBPs uniformity.^15^ Some publications have shown that the beam spot size adjustments can be based on the steepness of the distal fall‐off and the peak/plateau ratio, both being quite sensitive to this parameter.^10^, ^13^ For the Mevion S250 proton system, we observed up to 2 mm increase in the distal width of the pristine peaks due 7 mm increase in the SS, especially for the small group (Fig. 7).

We found that BA, OA, and SO significantly decreased the flatness and symmetry of the profiles (Figs. 8, 9, 10). Up to 2.0° tilt of proton beam resulted in nonsignificant effect on the D90% (<1 mm), while variations in the peak‐to‐plateau ratio were significant (Fig. 8). For the Mevion S250, the slope of the lexan and lead layers of second scatter are greater near its central axis.^2^ Therefore, slight changes in the beam SO (3 mm) produced significant uncertainty on the shape of the profiles, for all the studied options. The minimum OA required to induce a 2% change in symmetry was roughly 25% greater for the large option than for the small configuration (Table 2). Moreover, flatness of the profile was more sensitive to SO and OA changes near the central axis than in the periphery of the field, especially for small options. For deep group, the 5 mm OA from the central axis can significantly change the symmetry of the profile (up to 7.5%), while for small group lesser changes of OA (4 mm) was needed to produce a same effect (Figs. 8 and 9). This results in another source of uncertainty that can be included in a reference library guiding clinical medical physicists and engineers to troubleshoot and repair the machine, and also to tune the beam parameters to within clinically acceptable levels. Therefore, the proton beam line and second scatter must be taken into account as a system to evaluate changes in the shape of the profiles. While for a proton system already in clinical use, determining reasonable tolerances in all the moving parts in the beamline can be difficult, some measurements, i.e. beam profile due to second scatter offset, during acceptance testing of a new system may be helpful for later Monte Carlo commissioning work.

In addition to the uncertainties studied in this work, physics constants of various materials used in the construction of the treatment nozzle may produce another source of uncertainty.^16^ Previous studies have shown a significant sensitivity in MC calculated dose distribution on the variations in properties of materials used in passive scattering proton therapy treatment nozzles.^15^ Based on the sensitivity study performed on IBA (Louvain‐la‐Neuve, Belgium), the authors have reported that slight changes in density of the materials of field shaping parts clearly influenced the range and uniformity of dose distribution.^15^

These results can be used to improve quality assurance procedure or speed up commissioning process, especially for the commissioning of MC models of clinical passively scattered proton beams. Commissioning a MC model of a passively scattered proton can be a more rigorous and difficult process than it is for more standard treatment planning softwares. Correlating the many adjustable simulation parameters of the nuzzle with measurable dose distributions can notably facilitate the commissioning process. Moreover, to speed up the quality assurance of this system our results are an effective means of relating nuzzle parameters to clinical measurements.

The RBE of proton therapy is a function of dose, tissue endpoint, and energy deposition characteristics.^4^ In this regard, the LET can be used to parameterize the latter for proton beams, by taking into account range uncertainties, for a given dose and biological endpoint.^3^, ^6^ It is known that, the LET distribution in a proton beam depends on the range,^25^ therefore, uncertainty associated with range may affect LET distribution. In this study, it was hypothesized that changing the beam parameters including; AE, ES, and SS, alters the LET_t_ and LET_d_ distribution for a given proton beam. Generally, it is known that LET_t_ and LET_d_ are less sensitive in the plateau region, however, both LETs are highly sensitive to variations in energy near the Bragg peak when proton energy become low.^3^, ^7^ It has been stated that, for the passive‐scattering unmodulated monoenergetic proton beams of 250 MeV at the Proton Therapy Center at Houston (PTCH), LET_t_ is between 0.45 and 5.95 keV.μm^–1^.^26^ For proton beamlet of 201 MeV, the maximum LET_t_ and LET_d_ were reported to be 10.4 and 15.3 keV.μm^–1^, respectively, significantly higher, as expected, than the values calculated in our work.^7^

In our study, considering the sensitivity study of the LET, it was found that in the peak region, depending on the option, both LET_t_ and LET_d_ were sensitive to changes of AE, ES, and SS (Fig. 12, 13, 14). However, compared to the LET_d_ which showed higher sensitivity in the plateau region, LET_t_ showed higher sensitivity in the peak (up to 1.8%).

According to the previous reports for biological dose calculations, LET_d_ is more appropriate than the LET_t_ at therapeutically relevant dose levels.^3^ On the other hand, the proton biology experiments have shown the role of LET in the plateau region for determining cell kill is small.^7^ Therefore, in line with previous recommendation of Guan et al.^3^, we recommend the use of LET_t_ in the dose plateau region due to its characteristics of continuous increase along beam path and lower sensitivity to beam uncertainties. It means that a spatially variant switch between the use of LET_t_ and LET_d_ to quantify the LET is recommended for biological studies.

Recently, the idea of adaptive treatment planning by LET painting has started its development in the framework of TPS.^4^ Based on this idea, for a passively scattered proton treatment plan, optimization algorithm can attempt to minimize the volume of normal tissues exposed to high LET_d_, resulting in reducing radiation‐induced toxicity.

## 6 CONCLUSIONS

This study presents a detailed sensitivity analysis of most important but often poorly specified beam parameters required for simulating a gantry‐mounted passively scattered proton system. Our results revealed the importance of these parameters specially those resulted in large effects on the physical dose distribution and/or LETs, i.e., average proton beam energy, initial energy spread, spot size, and offset from the central axis. The findings can be used as a useful tool when quality assurance of this system. Moreover, the sensitivity analysis can also be used to aid machine design for determining reasonable tolerances in all the moving parts in the beamline. The simulation results from the sensitivity analysis can be utilized to construct a reference library to guide troubleshooting and repairing for the machine as well.
